# Quantitative prediction error analysis to investigate predictive performance under predictor measurement heterogeneity at model implementation

**DOI:** 10.1186/s41512-022-00121-1

**Published:** 2022-04-07

**Authors:** Kim Luijken, Jia Song, Rolf H. H. Groenwold

**Affiliations:** 1 Department of Clinical Epidemiology, Leiden University Medical Center, Leiden, the Netherlands; 2 Department of Biomedical Data Sciences, Leiden University Medical Center, Leiden, the Netherlands

**Keywords:** Prognostic model, Measurement heterogeneity, External validation, Calibration

## Abstract

**Background:**

When a predictor variable is measured in similar ways at the derivation and validation setting of a prognostic prediction model, yet both differ from the intended use of the model in practice (i.e., “predictor measurement heterogeneity”), performance of the model at implementation needs to be inferred. This study proposed an analysis to quantify the impact of anticipated predictor measurement heterogeneity.

**Methods:**

A simulation study was conducted to assess the impact of predictor measurement heterogeneity across validation and implementation setting in time-to-event outcome data. The use of the quantitative prediction error analysis was illustrated using an example of predicting the 6-year risk of developing type 2 diabetes with heterogeneity in measurement of the predictor body mass index.

**Results:**

In the simulation study, calibration-in-the-large of prediction models was poor and overall accuracy was reduced in all scenarios of predictor measurement heterogeneity. Model discrimination decreased with increasing random predictor measurement heterogeneity.

**Conclusions:**

Heterogeneity of predictor measurements across settings of validation and implementation reduced predictive performance at implementation of prognostic models with a time-to-event outcome. When validating a prognostic model, the targeted clinical setting needs to be considered and analyses can be conducted to quantify the impact of anticipated predictor measurement heterogeneity on model performance at implementation.

**Supplementary Information:**

The online version contains supplementary material available at 10.1186/s41512-022-00121-1.

## Background

Clinical prediction models for prognosis aim to provide predictions of an outcome for individuals who have not been part of the modelling process [1–5]. The quantity that a clinical prediction model targets is defined by specifying the outcome, (candidate) predictors, population, setting, time of prediction, and prediction horizon as specifically as possible [6]. When the research setting does not correspond to the intended setting of application in clinical practice [7, 8] or when modelling strategies are inappropriate [9, 10], the predictive performance of a prognostic model may be suboptimal at implementation.

One reason for suboptimal predictive performance of a model at implementation are differences in predictor measurement procedures between model development and implementation in practice [7, 11]. When discrepancies in predictor measurement procedures impact the performance of a clinical prediction model, this is referred to as *predictor measurement heterogeneity* [12]. The impact of predictor measurement heterogeneity on predictive performance at external validation has been quantified for models of binary outcome data [11–14] and illustrated in empirical datasets for logistic regression diagnostic prediction models [11, 15]. However, the step towards model implementation in a target population has not been studied yet. The impact of predictor measurement heterogeneity in time-to-event data has not received adequate attention either.

Previous studies on predictor measurement heterogeneity defined heterogeneous predictor measurements using measurement error models [16, 17] by varying the degree of measurement error across settings of derivation and validation [11, 12, 15]. Measurement error in predictor variables in regression analysis is known to result in biased estimates of regression coefficients [17, 18]. For instance, non-differential random measurement error in a continuous predictor attenuates the regression coefficient for that variable. However, a prediction model that includes a predictor measured with error can still yield valid predictions in the setting it was derived in. Predictions based on error-prone measurements can also be correct in external settings, i.e., at validation or implementation, provided that the degree of error in the predictor measurement is similar to that of the derivation setting. When the measurement of the predictor is subject to different amounts of error compared to the derivation setting, this could hamper the transportability of the model.

Methods to correct for measurement error can be used to obtain prediction models with unbiased estimates of regression coefficients when predictors are measured with error [17–20]. However, since measurement error correction is not often performed in prediction studies [21] and practically infeasible at implementation, we focus on the impact of differences in degree of measurement error across settings on predictive performance of models that are uncorrected for measurement error in (one of) the predictors.

In the current study, we suggest an approach to anticipate the impact of predictor measurement heterogeneity on a prognostic model when it is implemented in clinical practice. We assess the impact of predictor measurement heterogeneity in time-to-event outcome data using large-sample simulations. We propose a quantitative prediction error analysis for validation studies that can be used to quantify the impact of anticipated predictor measurement heterogeneity in one of the predictors. This is illustrated using an example of a model predicting the 6-year risk of developing type 2 diabetes.

## Predictor measurement heterogeneity

**Fig. 1:**
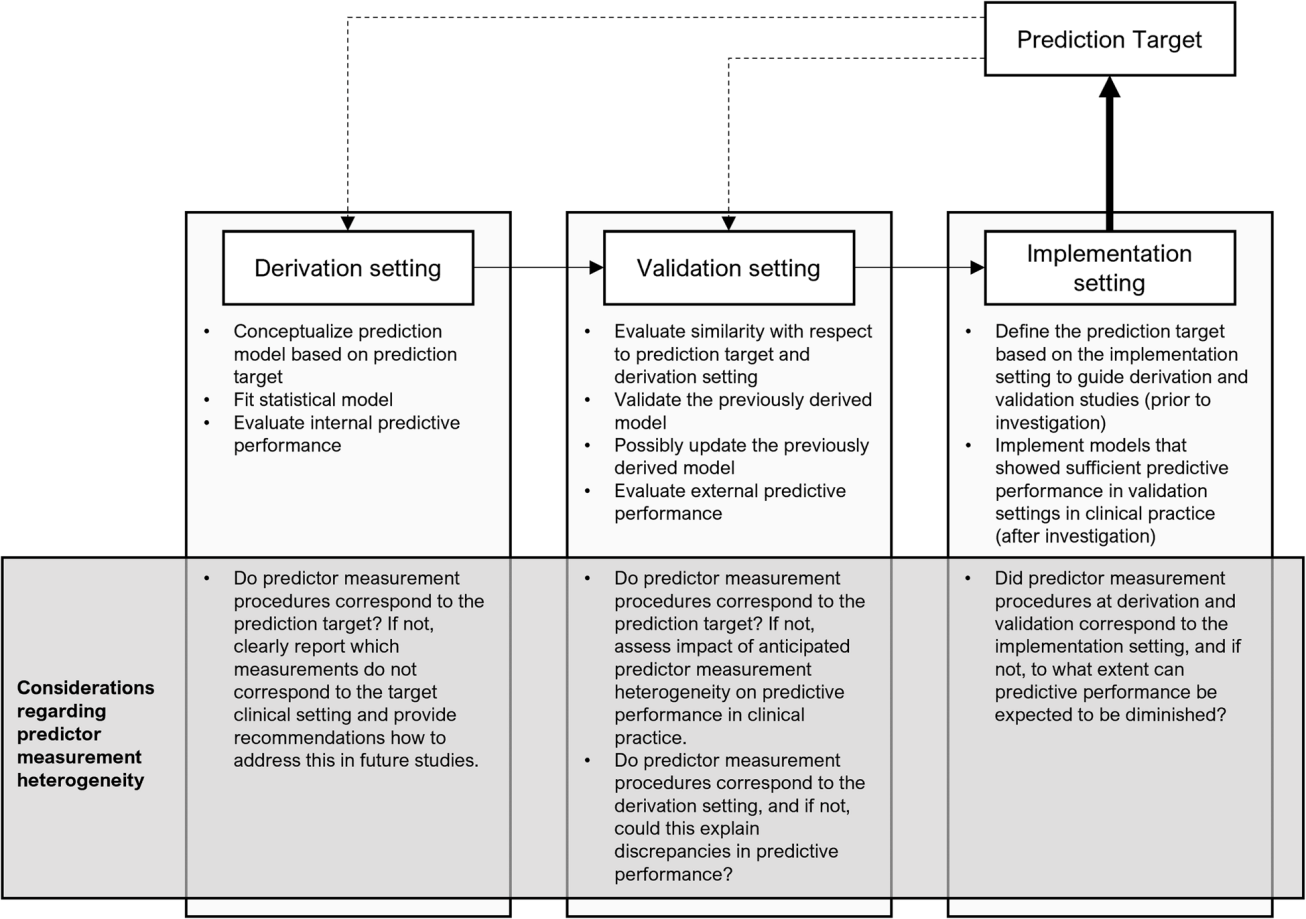
An overview of the derivation, validation, and implementation setting of a prognostic model, highlighting considerations regarding predictor measurement heterogeneity. Note that “impact analysis” research is a phase between validation and implementation that is not addressed in this diagram. A prediction target is defined by specifying the target population, setting, outcome, (candidate) predictors, time of prediction, and prediction horizon as specifically as possible

For a prognostic model to provide correct predictions of an outcome in a clinical setting, several phases of model development should be considered, which is outlined in Fig. 1 [5, 22–24]. Ideally, a prognostic model is derived using data that correspond to the targeted implementation setting (derivation setting) [25, 26]. Predictive performance is typically evaluated by measures of apparent performance and measures of performance after internal validation of the model, i.e., after correcting for optimism about the performance [27, 28]. When the internal predictive performance of the model is sufficient, its performance can be investigated using external (validation)data [29, 30], preferably multiple times [31–33] (validation setting). When predictive performance at external validation is sufficient, implementation of the model in clinical practice could be considered (implementation setting), advisably after performing an impact analysis [34, 35].

One aspect to consider in all phases of development of a prognostic model is predictor measurement heterogeneity, indicated in the grey box in Fig. 1. Procedures to collect and measure predictor data for derivation and validation studies ideally correspond to the future implementation setting. When predictor measurement procedures at derivation and/or validation deviate from the predictor measurement procedure used in clinical practice, this can affect the predictive performance at implementation.

## Simulation study

We performed a simulation study to investigate the impact of predictor measurement heterogeneity across validation and implementation setting on out-of-sample predictive performance of a survival model developed and validated in time-to-event outcome data. We assumed that all other possible sources of discrepancy in predictive performance are not present, e.g., there are no differences in outcome prevalence and treatment assignment policy, there is no overfitting with respect to the derivation data, and the prognostic model is correctly specified in terms of functional form and included interactions. We used (very) large samples (*n* = 1,000,000) to minimize the role of random simulation error.

### Design of simulation study

Online Supplement 1 contains a detailed description of the simulation study. The main aspects of the design of the simulations study are described below and reported according to previous recommendations [36].

#### Data-generating mechanism

We simulated derivation, validation, and implementation datasets with 1,000,000 observations containing a continuous predictor variable *X* from a standard normal distribution. A time-to-event outcome was simulated for each subject so that outcomes followed a Cox-exponential model, using methods described by Bender and colleagues [37] (see Table 1 for simulation parameters). We generated datasets without censoring (median survival time *t* = 6.6). Additionally, datasets with administrative censoring after *t* = 15 (74% event fraction, median survival time 6.6) and with random censoring (69% event fraction, median survival time *t* = 5.6) were generated.

**Table 1:** Simulation parameters

Parameter	Value
Baseline hazard of an event	0.1
Conditional hazard ratio for association predictor *X* and survival times	2
Time point of administrative censoring	15
Baseline hazard of censoring	0.01
Conditional hazard ratio for association between random variable for censoring and censoring times	3
Mean of predictor *X* and random variable for censoring	0
Variance of predictor *X* and random variable for censoring	1
Predictor *W* at implementation*	
ψ	− 0.3 to 0.3
*θ*	0.5 to 2
*σ*_*ε*_	0 to √2

*At implementation, a different measurement of predictor *X* was available, denoted measurement *W.* The connection between *X* and *W* was defined using the following measurement heterogeneity model: $\mathbbm{E}(W)=\psi +\theta \mathbbm{E}(X)+\epsilon,$ where $\epsilon \sim \mathcal{N}\left(0,{\sigma}_{\epsilon}^2\right)$, and where ψ denotes an additive shift in *W* with respect to *X*, *θ* denotes a multiplicative linear association between *W* and *X*, and *σ*_*ε*_ denotes random deviations from *X*

At implementation, a different measurement of predictor *X* was available, denoted *W*. Predictor measurement heterogeneity across validation and implementation setting was recreated using measurement error models, similar to [12]. The mean difference between *X* and *W* was denoted ψ (additive systematic measurement heterogeneity), the linear association between *X* and *W* was denoted *θ* (multiplicative systematic measurement heterogeneity), and the variance introduced by random deviations from *X* was denoted${\sigma}_{\varepsilon}^2$, where non-zero values of ${\sigma}_{\varepsilon}^2$ reflect that measurement *W* is less precise than *X* (random measurement heterogeneity).

In total, 162 scenarios were evaluated (27 scenarios of predictor measurement heterogeneity, for 2 different models under 3 different censoring mechanisms).

#### Prediction target

The prediction target was defined as obtaining correct predictions of the outcome risk at time point *t* = 6.5 conditional on predictor measurement *W* measured at the time of prediction (i.e., at *t* = 0).

#### Methods

A parametric exponential survival model and a semi-parametric Cox regression model were fitted in the derivation dataset. Although a prognostic model is typically internally validated before performing external validation [1, 27], we did not perform an internal validation since issues of overfitting were expected to be negligible due to the large number of events relative to the number of predictors. The prognostic model was externally validated at time *t* = 6.5 (around median survival time) under predictor measurement homogeneity in an independent (validation) dataset. Predictor measurement homogeneity refers to the situation in which predictors are measured in the same way at derivation and validation. Furthermore, the predictive performance of the prognostic model was investigated in various implementation settings under predictor measurement heterogeneity. Notably, the models were validated under predictor measurement heterogeneity as-is, without correcting for differences in measurement procedures. In each simulation scenario, the different steps outlined here were performed once.

#### Performance metrics

Predictive performance was evaluated at *t* = 6.5, i.e., approximately at the median survival time. Calibration of the model on average, or “calibration in the large” [38, 39], was evaluated by the ratio of the observed marginal survival at *t* = 6.5 (obtained through a Kaplan-Meier curve) versus the predicted marginal survival at *t* = 6.5 (obtained by averaging predicted survival at *t* = 6.5 of each observation), denoted the observed/expected ratio (O/E ratio). Discrimination was evaluated by the cumulative-dynamic time-dependent area under the receiver operating characteristic curve AUC(*t*) [40–42]. Overall accuracy was evaluated by the index of prediction accuracy at *t* = 6.5, IPA(*t*), which equals a Brier score [43] at *t* = 6.5 that is benchmarked to a null model ignoring all patient specific information and simply predicts the empirical prevalence to each patient [44]. A perfect model has an IPA of 1, a non-informative model has an IPA of 0 and a negative IPA indicates a harmful model.

#### Software

The simulation study was performed using R statistical software version 3.6.3 [45]. The simulation code is available from https://github.com/KLuijken/PMH_Survival.

### Results of simulation study

Predictor measurement heterogeneity affected predictive performance at implementation. In all scenarios of predictor measurement heterogeneity, the prognostic models were miscalibrated in the large (range O/E ratio 0.89 to 1.19, compared to 1.00 under predictor measurement homogeneity), and overall accuracy was reduced (range IPA(6.5) − 0.17 to 0.17, compared to 0.17 under predictor measurement homogeneity). The AUC(6.5) (range 0.58 to 0.74, compared to 0.74 under predictor measurement homogeneity) was particularly affected by random predictor measurement heterogeneity. We present results for the Cox regression model under no censoring only. The impact on the measures of predictive performance under administrative and uninformative (random) censoring and for the parametric exponential survival model was similar (data in Online Supplement 1, Section 3).

**Fig. 2:**
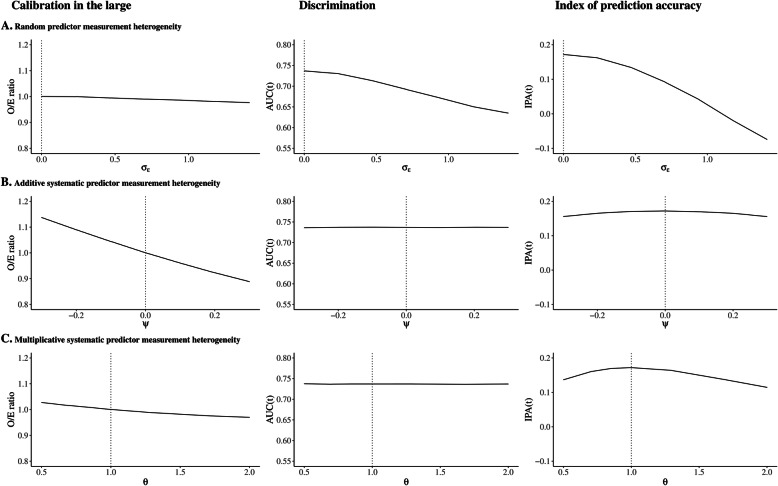
Measures of predictive performance under predictor measurement heterogeneity between validation and implementation setting. Results shown for random predictor measurement only (**A**), additive systematic predictor measurement only (**B**), and multiplicative systematic predictor measurement heterogeneity only (**C**). The vertical dashed line indicates predictor measurement homogeneity between validation and implementation setting. The *x*-axes show measurement heterogeneity parameters describing the predictor measurement at implementation relative to the predictor measurement at validation, where *σ*_*ε*_ denotes random deviations from the measurement at validation, ψ denotes an additive shift with respect to the measurement at validation, and *θ* denotes a systematic multiplicative association with the measurement at validation. Note that additional simulation scenarios were run to smooth the plots

As measurement procedure *W* contained more random variability compared to *X*, i.e., a case of random measurement heterogeneity, *σ*_*ε*_ = 0 at validation and *σ*_*ε*_ > 0 at implementation, the O/E ratio moved slightly under 1 (Fig. 2A). The AUC(6.5) and IPA(6.5) decreased as random measurement heterogeneity increased.

Additive systematic measurement heterogeneity, i.e., ψ = 0 at validation and ψ ≠ 0 at implementation, affected the calibration-in-the-large coefficient at implementation, but minimally affected the AUC(6.5), and IPA(6.5) at implementation (Fig. 2B). When measurement procedure *W* at implementation provided a systematically higher value of the predictor compared to measurement procedure *X* at validation, i.e., ψ > 0, this resulted in overestimation of the average outcome incidence at implementation, and the O/E ratio < 1.

Multiplicative systematic measurement heterogeneity, i.e., *θ* = 1 at validation and *θ* ≠ 1 at implementation, yielded an O/E ratio < 1 in case *θ* > 1 (Fig. 2C). Multiplicative systematic measurement heterogeneity minimally affected the AUC(6.5) in absence of additive systematic and random measurement heterogeneity. As *θ* was further from 1, the IPA(6.5) at implementation decreased, indicating lower overall accuracy.

Combined random, additive systematic, and/or multiplicative systematic predictor measurement heterogeneity sometimes reinforced or cancelled out effects on predictive performance (see Online Supplement 1, Section 3).

## Illustration of quantitative prediction error analysis

We describe an analysis that quantifies the impact of anticipated predictor measurement heterogeneity between the validation and implementation setting. The analysis is illustrated by validation of a prognostic model predicting the 6-year risk of developing type 2 diabetes. The section “Motivating example” describes validation and updating of the model in an example validation dataset. The hypothetical step to implementation is described in the section “Quantifying the impact of anticipated predictor measurement heterogeneity between validation and implementation setting” by means of a seven-step quantitative prediction error analysis (Table 2). The proposed analysis can be performed to assess the impact of anticipated heterogeneity in measurement of one of the predictors across settings of validation and implementation. A detailed description including analysis code can be found in Online Supplement 2.

**Table 2:** Quantitative prediction error analysis to quantify the impact of anticipated predictor measurement heterogeneity at implementation of a prognostic model in clinical practice (details in section “Quantifying the impact of anticipated predictor measurement heterogeneity between validation and implementation setting” of the main text)

1. State the prediction target. 2. Report whether predictor measurement procedures in the validation setting correspond to those at implementation. 3. Identify one predictor that is expected to be measured using a different procedure in the implementation setting than in the validation setting. 4. Define a model for the relation between the measurement in the validation study and its equivalent in the implementation setting. 5. Perform a literature search to establish a range for the size of the possible parameters of predictor measurement heterogeneity. 6. Simulate the scenarios of anticipated measurement heterogeneity to assess the possible impact on predictive performance. 7. Report the impact of anticipated predictor measurement heterogeneity on predictive performance at implementation in clinical practice.	

### Motivating example

Zhang and colleagues derived a prognostic model for the 6-year risk of developing type 2 diabetes from the predictors age, body mass index (BMI), triglyceride, and fasting plasma glucose at the time of prediction [46]. In the derivation study (*n* = 11,564), the incidence density rate was 9.57/1000 person years (659 events in total) [46]. Performance of the prediction model was measured in terms of the area under the receiver operating characteristic curve (not further specified), which was equal to 0.77 (95% CI, 0.76 to 0.78). As a small remark, the reported regression coefficient of fasting plasma glucose did not equal the logarithm of the corresponding hazard ratio, which we assumed was a typo. We used the reported regression coefficient in model validation, as this was the focus in the main text of Zhang and colleagues.

The example dataset for validation was a publicly available dataset containing information about 15,464 individuals who participated in a medical examination program at the Murakami Memorial Hospital from 2004 to 2015, made available alongside a study by Okamura and colleagues [47]. We considered the validation sample to be similar to the derivation setting (see Online Supplement 2 for a more detailed comparison of the derivation and validation setting). BMI was reported to be measured at medical examination; we assumed it was computed from scale and measuring-tape measurements and thus assumed no predictor measurement heterogeneity across derivation and validation setting. We censored follow-up after 6 years and assumed censoring before that time was non-informative. The incidence density rate was 2.84/1000 person years (192 events in total), event times ranged from 285 to 2191 days, and censoring times ranged from 164 to 2192 days.

We evaluated predictive performance at 6 years using the performance measures described in our simulation study. At validation, the calibration-in-the-large O/E ratio was 0.47 (95% CI, 0.41 to 0.54), indicating that predicted risks were overestimated on average. The AUC(6 years) was 0.89 (95% CI, 0.85 to 0.89), indicating good discriminatory performance of the model. The IPA(6 years) was 0.02 (95% CI, 0.01 to 0.03), indicating low overall accuracy of the model.

Given the suboptimal calibration of the model and the difference in outcome incidence between derivation and validation setting, we updated the model by recalibrating the baseline survival for being diabetes free using an offset for the linear predictor [48]. Predictive performance of the model after updating was as follows: the calibration-in-the-large O/E ratio was 1.02 (95% CI, 0.90 to 1.18), the AUC(6 years) was 0.87 (95% CI, 0.85 to 0.89), and the IPA(6 years) was 0.04 (95% CI, 0.04 to 0.05).

### Quantifying the impact of anticipated predictor measurement heterogeneity between validation and implementation setting

Seven steps are described to perform a quantitative prediction error analysis in a prognostic model validation study to assess the impact of anticipated heterogeneity in measurement (Table 2). For the example described above, we anticipate that BMI will be calculated based on measurement of self-reported height and weight at implementation, instead of tape and scale measures at validation.

First, the prediction target is stated. In this example, the prediction target would be the 6-year risk of developing type 2 diabetes in Asian adults presenting for preventive medical examination by measurements of age, BMI, triglyceride, and fasting plasma glucose at the time of prediction. Incident diabetes is defined as HbA1c ≥ 6.5% (48 mmol/mol) in two test results, measured using a standardized method [49]. Age is measured in years, BMI is calculated from self-reported weight and height, triglyceride is measured according to standards of the National Institute of Standards and Technology [50], and fasting plasma glucose is measured using a standardized method [51, 52]. Details on procedures to measure HbA1c, triglyceride, and fasting plasma glucose are omitted here for brevity, but are ideally described in more detail in an empirical study [7]. Treatment assignment policy was assumed to be similar in the research settings compared to the target clinical setting and interventions such as diet were not modeled explicitly (i.e., ignore-treatment strategy [53]).

Second, it is described whether predictor measurement procedures in the validation setting correspond to those that will be used at implementation. Measurements of age, triglyceride, and fasting plasma glucose roughly correspond to the target predictor measurement procedures. However, the validation study measured BMI during medical examination of a patient, which differs from self-reported measurements defined in the prediction target.

Third, a predictor is identified that is expected to be measured differently (e.g., using a different procedure) in the implementation setting compared to the validation setting. In the example, measurement heterogeneity was expected to be largest for the predictor BMI.

Fourth, a model for the relation between the measurement of BMI in the validation study, *BMI*_*val*_, and in the implementation setting, *BMI*_*imp*_, is defined, e.g.,:

$$ {BMI}_{imp}=\psi +\theta {BMI}_{val}+\varepsilon, $$

where $\varepsilon \sim \mathcal{N}\Big(0,{\sigma}_{\varepsilon}^2$), and ψ ≠ 0 indicates that measurements of BMI in the implementation setting are systematically additively shifted with respect to BMI in the validation study, *θ* ≠ 1 indicates measurements of BMI in the implementation setting are systematically multiplicatively altered with respect to BMI in the validation study, and *σ*_*ε*_ > 0 indicates measurements of BMI in the implementation setting contain more random variation relative to BMI in the validation study.

Fifth, the range is specified for the parameter values of the model for the anticipated predictor measurement heterogeneity, as defined in Step 4. A literature search was performed to identify studies describing measurement error in BMI. Informed by studies comparing measured and self-reported BMI values [54–58], the range of measurement error parameters was specified as − 1 to 0 for ψ, 0.9 to 1 for *θ*, and 0 to 1.5 for *σ*_*ε*_. In general, we advise to use terms like “measurement error,” “validation study,” and the measurement procedures to search for relevant literature. Of note, the term “validation study” has a different meaning in prediction literature compared to measurement error literature. In prediction modelling research, a validation study refers to a study that evaluates the predictive performance of an existing prediction model. In measurement error literature, a validation study refers to a study that investigates the relation between a perfect (error-free) measurement and its (error-prone) proxy measurement, usually in a subset of individuals included in the study [17]. In the current study, we thus far used the term “validation study” according to the prediction literature.

**Fig. 3:**
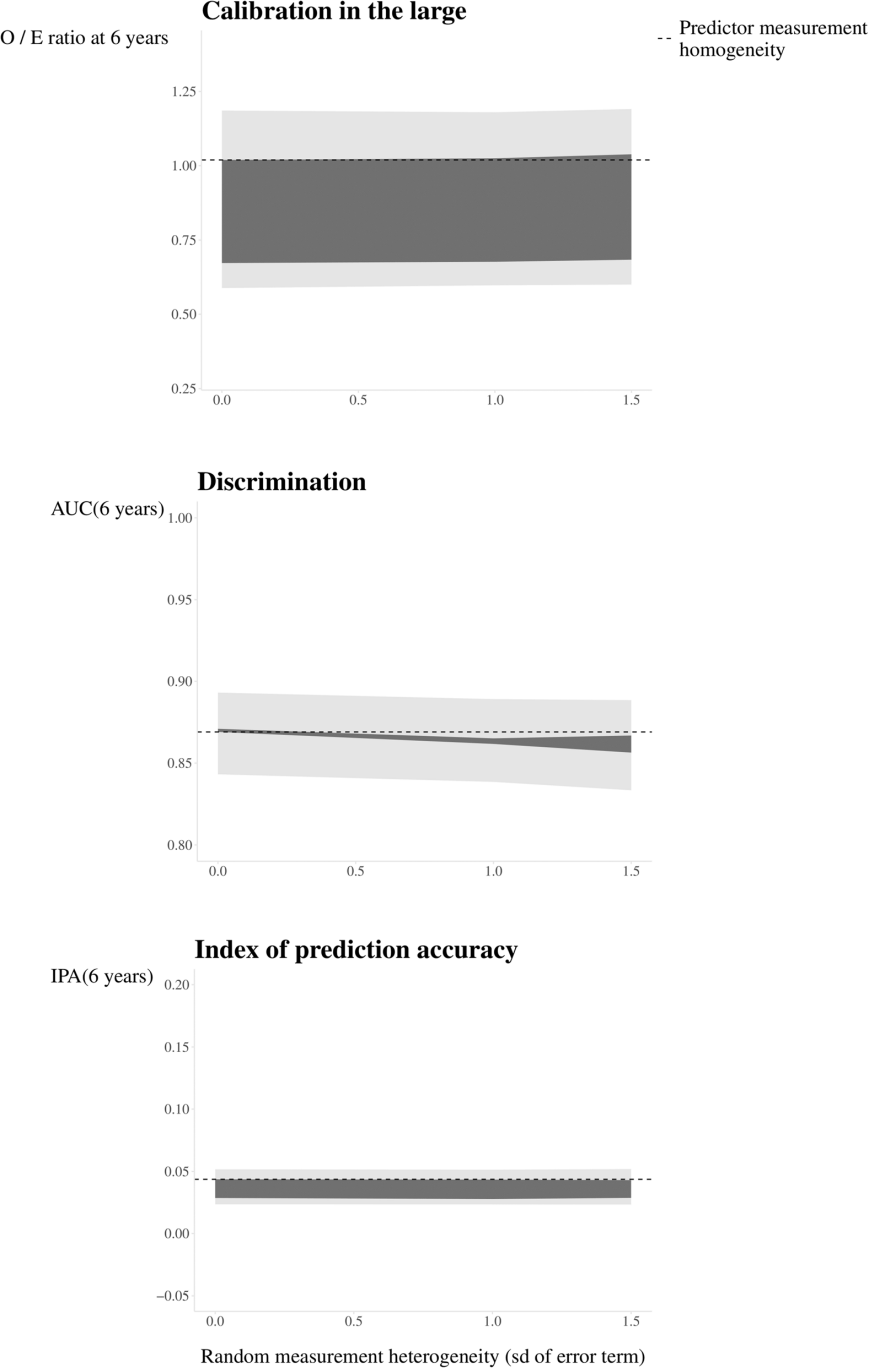
Impact of anticipated heterogeneity in measurement of the predictor body mass index on measures of predictive performance at implementation of a model to predict the 6-year risk of developing diabetes type 2. The dotted line indicates predictive performance under predictor measurement homogeneity. Dark grey indicates the impact within the range of specified predictor measurement heterogeneity and light grey indicates the range of 95% CIs from 500 bootstrap resamples. Random predictor measurement heterogeneity is presented on the *x*-axis, and performance measures are marginalized over scenarios of additive and multiplicative systematic predictor measurement heterogeneity

Sixth, the scenarios of anticipated measurement heterogeneity can be investigated using statistical simulations to assess the possible impact on predictive performance. Briefly, we plugged the values found in Step 5 into the model specified in Step 4 to generate measurements of BMI that can be anticipated in the implementation setting in participants otherwise similar to the validation sample. We evaluated the O/E ratio for calibration in the large, AUC(6 years), and IPA(6 years) under the scenarios of measurement heterogeneity in BMI (see Online Supplement 2) and plotted the outcomes (Fig. 3).

Seventh, the impact of anticipated predictor measurement heterogeneity on predictive performance in the implementation setting can be reported in a validation study, accompanied by a description of Steps 1–6. Figure 3 illustrates the range of the O/E ratio at 6 years, AUC(6 years), and IPA(6 years) under the anticipated measurement heterogeneity of BMI across validation and implementation setting. The findings suggest that model discrimination and overall accuracy are likely minimally affected by the change in measurement of BMI. However, with increasing differences in BMI measurement, model miscalibration increases and predicted risks are more likely to be overestimated on average.

Possible consequences of this finding may be either to recommend collecting data on BMI using scale and measuring-tape measures when the model is used in clinical practice to predict 6-year risk of developing diabetes or to update the current prediction model using self-reported measures of BMI before implementing it in clinical practice. In the current example, it is likely not worthwhile to perform another study in which data on BMI is collected using self-reported measures rather than measuring BMI using a scale and measuring tape to update the coefficient for BMI. One reason for this is that it is unlikely that clinical decisions will change when the 6-year risk prediction of developing diabetes is overestimated, in particular because the average predicted risk is around 1% and predicted risks are overestimated around 1.5 times compared to observed risks in extreme cases of predictor measurement heterogeneity of BMI.

## Discussion

Our simulations indicated that predictor measurement heterogeneity across the validation and implementation setting of a prognostic model can substantially affect predictive performance at implementation. We illustrated how a quantitative prediction error analysis can be applied in validation studies to quantify the impact of anticipated dissimilar predictor measurements in the clinical target setting on predictive performance. Based on this analysis, a validation study can inform readers about the degree to which anticipated predictor measurement heterogeneity affects predictive performance when the model is implemented in clinical practice.

The rationale for the quantitative prediction error analysis was analogous to the quantitative bias analysis framework by Lash and colleagues, which can be applied to estimate the direction, magnitude, and uncertainty from systematic errors affecting studies of causal inference [59, 60]. While Lash and colleagues encourage researchers to address multiple sources of bias [59], we focused on a single source of heterogeneity across settings that can affect performance of a clinical prediction model. We focused on non-differential systematic and random measurement heterogeneity in a single predictor, where the clinical implementation setting contained more measurement variance compared to the validation setting. Future work could extend these quantitative prediction analyses to non-differential measurement heterogeneity, to situations where the clinical implementation setting contains less measurement variance compared to the validation setting—for instance through methods analogous to the simulation-extrapolation method (SIMEX) [61, 62]—and to models that take into account correlations of measurement heterogeneity structures when multiple predictors are expected to be measured heterogeneously across validation and implementation setting. Additionally, other sources of heterogeneity across settings that can affect performance of a clinical prediction model can be added to the quantitative prediction error analysis, such as heterogeneity in event rate, heterogeneity in outcome measurement procedures, and heterogeneity in treatment-assignment policies during follow-up.

The example of predicting the risk of developing type 2 diabetes illustrated the impact anticipated measurement heterogeneity in the predictor BMI. Notably, the magnitude of the impact of anticipated measurement heterogeneity depends on whether the linear predictor is centered to the validation data. While many functionalities in R statistical software [45] center the linear predictor by default, centering is likely uncommon in clinical practice and obviously decreases the impact of predictor measurement heterogeneity on predictive performance. A limitation of our example is that measurement heterogeneity was only considered in a single predictor, whereas the predictor fasting plasma glucose can potentially be measured heterogeneously across settings as well, in particular because fasting instructions and adherence to instructions may differ across settings. Taking this into account requires consideration of the duration of fasting relative to the timing of the plasma glucose measurement [21]. Modelling the functional form of fasting plasma glucose or another (circadian) fluctuating hormone or biomarker over time to assess the impact in heterogeneity of measurement timings across time would be an interesting topic for future research.

As a limitation to our study, the simulations lacked a comparison of predictive performance under predictor measurement heterogeneity of models that were validated as-is to models that were corrected for measurement error in the predictions. We focused on implementation of models as-is because this is commonly done in practice, but that comparison would have provided additional insights on predictive performance under predictor measurement heterogeneity and could be the topic of future research. Additionally, implementation of the quantitative prediction error analysis may be hampered because literature informing the choice of measurement error parameters (Step 5) may be limited. When no information is available about predictor measurement structures in an implementation setting of interest, it might be helpful to set up a (measurement heterogeneity) validation study to estimate the predictor measurement heterogeneity parameters directly [17]. This may be an alternative approach to anticipate the performance of a prognostic model in a particular setting that is likely less cumbersome than conducting a prediction validation study in the implementation setting.

## Conclusions

Heterogeneity of predictor measurements across settings of validation and implementation had a substantial influence on predictive performance at implementation of prognostic models with a time-to-event outcome. Data for derivation and validation of prognostic models are collected ideally using procedures that match the target clinical setting (i.e., how and where the model will be implemented in clinical practice). When this is infeasible, a quantitative prediction error analysis provides an analytical approach to quantify the anticipated impact of the discrepancies between available research data and clinical practice.

## Supplementary information

**Additional file 1.** Simulation study assessing the impact of predictor measurement heterogeneity across validation and implementation setting in time-to-event outcome data.

**Additional file 2.** Sensitivity analysis assessing the impact of anticipated predictor measurement heterogeneity across validation and implementation setting in time-to-event outcome data. An example on prediction of incident diabetes type 2.

## Data Availability

The dataset illustrating the quantitative prediction error analysis in this article is available in the Dryad repository, 10.5061/dryad.8q0p192
