# Supplementary material for: Quantitative prediction error analysis to investigate predictive performance under predictor measurement heterogeneity at model implementation
Source: Diagn Progn Res. 2022 Apr 7;6:7. doi: 10.1186/s41512-022-00121-1 (PMC8988417; doi:10.1186/s41512-022-00121-1)
Supplement: Supplementary file 1 — Additional file 1. Simulation study assessing the impact of predictor measurement heterogeneity across validation and implementation setting in time-to-event outcome data. [file 41512_2022_121_MOESM1_ESM.pdf]

Simulation study assessing the impact of predictor measurement heterogeneity across validation and implementation setting in time-to-event outcome data

Supplementary File 1

Kim Luijken
Department of Clinical Epidemiology
Leiden University Medical Center

k.luijken@lumc.nl

Jia Song
Department of Clinical Epidemiology
Leiden University Medical Center

Rolf Groenwold
Department of Clinical Epidemiology
Leiden University Medical Center

December 22, 2021

Content

This Supplementary File accompanies the manuscript "Quantitative prediction error analysis to investigate predictive performance under predictor measurement heterogeneity at model implementation" by Kim Luijken, Jia Song, and Rolf Groenwold. It contains a description of the simulation study described in Section 3 of the main text.

1 Design of simulation study and implementation in R code

1.1 Aim

We performed a simulation study to illustrate the impact of predictor measurement heterogeneity across validation and implementation setting on out-of-sample predictive performance of a survival model developed and validated in time-to-event outcome data, given that all other possible sources of discrepancy in predictive performance are not present, i.e., when there are no differences in outcome prevalence and treatment assignment policy, when there is no overfitting with respect to the derivation data, the prognostic model is correctly specified in terms of functional form and included interactions. We used (very) large samples (n = 1,000,000) to minimize the role of random simulation error.

1.2 Data-generating mechanism

1.2.1 Time-to-event data

We simulated derivation, validation, and implementation data sets with 1,000,000 observations containing a continuous predictor variable X from a standard normal distribution, which one can think of as a linear predictor or risk score that summarizes the information of a set of predictor variables. We then simulated a time-to-event outcome, i.e., an event time T and and indicator variable Y denoting the outcome event of interest, for each subject so that outcomes followed a Cox-exponential model, using methods described by Bender and colleagues [1]. The association between X and T equaled log(2), and the baseline hazard equaled 0.1. We generated data sets without censoring (median survival time $t = 6.5$).

```
# Rcode corresponding to file ./R/dgm.R
n_obs      <- 1000000


X          <- rnorm( n_obs, mean = 0, sd = 1)
U          <- runif( n_obs, min = 0, max = 1)
time_event <- -log( U) / ( 0.1 * exp( X * log( 2)))
event      <- rep( 1, times = n_obs)
```

Additionally, to mimic administrative censoring (censoring scenario 1), survival times were censored after $t = 15$ (74% event fraction, median survival time 6.5).

```
# Rcode censoring scenario 1

event                   <- as.integer( time_event < 15)
time_event[ event == 0] <- 15
```

In censoring scenario 2, random censoring of survival times was applied by simulating censoring times, where the association between a ‘censoring predictor’ and the censoring times equals log(3) and the baseline hazard equals 0.01 (69% event fraction, median survival time $t = 5.5$).

```
# Rcode censoring scenario 2

U_cens      <- runif( n_obs, min = 0, max = 1)
X_cens      <- rnorm( n_obs, mean = 0, sd = 1)
time_cens   <- -log( U_cens) / ( 0.01 * exp( X_cens * log( 3)))

time_event_random  <- pmin( time_event, time_cens)
event              <- as.integer( time_event_random == time_event)
```

1.2.2 Predictor measurement heterogeneity

Predictor measurement heterogeneity was recreated using measurement error models, similar to [2]. To distinguish different measurements of the same predictor, we denoted an exact measurement of the predictor (e.g., bodyweight measured on a scale) by X and a pragmatic measurement (e.g., self-reported weight) by W. Let ψ reflect the mean difference between X and W, let θ indicate the linear association between measurement

X and W, and let σ_ϵ^2 reflect the variance introduced by random deviations in the measurement process of W, where a larger σ_ϵ^2 indicates that measurement W is less precise. We defined a general model of measurement heterogeneity for continuous predictors in line with existing measurement error literature[3, 4]. Assuming that the relation between X and W is linear and additive, the association between X and W can be described as

$$\mathbb{E}(W) = \psi + \theta\mathbb{E}(X) + \epsilon, \tag{1}$$

where $\epsilon \sim \mathcal{N}(0, \sigma_\epsilon^2)$ is independent of X, T, and Y. In case of $\psi = 0, \theta = 1$, and $\sigma_\epsilon^2 = 0$, there is no difference between the predictor measurement procedures across the validation study and target clinical setting or *predictor measurement homogeneity*, i.e., $\mathbb{E}(W) = \mathbb{E}(X)$.

We assumed W to be a surrogate measurement of X, or non-differential measurement error, meaning that the contribution from the observed W is not informative for the survival time given X. Furthermore, we assume ϵ_i to be independent from X, or homoscedastic measurement error.

The derivation data and validation data contained measurements of predictor X, i.e., there was predictor measurement homogeneity across derivation and validation setting. The implementation setting contained measurements of predictor W, i.e., there was predictor measurement heterogeneity across validation and implementation setting. The parameters of measurement error model (1) were varied to recreate 27 scenarios (3 x 3 x 3) of predictor measurement heterogeneity.

```
# Simulation parameter values to recreate predictor measurement heterogeneity

psi           <- c( -0.3, 0 , 0.3)
theta         <- c( 0.5, 1, 2)
sigma_epsilon <- c( 0, 0.5*sqrt(2), sqrt(2))
```

1.3 Prediction target

The prediction target was defined as obtaining correct predictions of the outcome risk at time point $t = 6.5$ conditional on predictor measurement W measured at moment of prediction (i.e., at $t = 0$).

1.4 Methods

Using the derivation data set, two survival models were fitted: a parametric exponential survival model and a semi-parametric Cox regression model.

```
# Rcode corresponding to file ./R/_targets.R

# derive survreg parametric model based on X
  mod_exp   <- rms::psm( survival::Surv( time_event, event) ~ X,
                         dist = "exponential",
                         data = data_der,
                         x = T, # return design matrix
                         y = T # return Surv() matrix
  )

# derive semi-parametric Cox model based on X
  mod_cox   <- survival::coxph( survival::Surv( time_event, event) ~ X,
                      data = data_der,
                      x = T,
                      y = T)
```

Although a prediction model is typically internally validated before performing external validation [5, 6], we did not perform an internal validation since issues of overfitting were expected to be negligible in a sample of 1,000,000 observations. The prediction model was externally validated in a validation data set at time $t =$ 6.5 (corresponding to the median survival time) under predictor measurement homogeneity. Furthermore, the prediction model was externally validated in various clinical implementation settings under predictor measurement heterogeneity.

Validating an parametric exponential survival model and a cox model in data under all 3 censoring mechanisms for all 27 scenarios of predictor measurement heterogeneity resulted in 162 scenarios.

1.5 Performance metrics

Predictive performance at $t = 6.5$ was evaluated in terms of calibration, discrimination, and overall accuracy. Calibration of the model on average, or 'calibration in the large' [7, 8] was evaluated by the ratio of the observed marginal survival at $t = 6.5$ (obtained through a Kaplan-Meier curve) versus the predicted marginal survival at $t = 6.5$ (obtained by averaging predicted survival at $t = 6.5$ of each observation), denoted the observed / expected ratio (O/E ratio).

```
# Rcode corresponding to file ./R/analysis.R

  # change predictor name W to X
  implementation_data <- data.frame( time_event = data_val$time_event,
                                     event = data_val$event,
                                     X = data_val$W)

  # linear predictor
  lp <- if( class( mod)[1] == "coxph"){
        predict( mod,
                 newdata = implementation_data,
                 type = "lp")}else{
                   -predict( mod,
                             newdata = implementation_data,
                             type = "lp")
                 }

  # take marginal predicted risk
  pred_risk <- 1 - mean( pec::predictSurvProb( mod,
                                               newdata = implementation_data,
                                               times = t_val))
  obs_risk <- 1 - summary( survfit( Surv( time_event, event) ~ 1,
                                    data = sim_data),
                           times= t_val)$surv

  cal_large <- obs_risk / pred_risk
```

Discrimination was evaluated by the cumulative-dynamic time-dependent area under the receiver operating characteristic curve AUC(t) [9–11].

```
# Rcode corresponding to file ./R/analysis.R

  # evaluate time-dependent cumulative c-statistic using timeROC package
  c_stat <- timeROC::timeROC( T = data_val$time_event,
                              delta = data_val$event,
                              cause = 1,
                              marker = lp, # linear predictor as above
                              times = t_val)$AUC[2]
```

Overall accuracy was evaluated by the index of prediction accuracy at $t = 6.5$, IPA(t), which equals a Brier score [12] at $t = 6.5$ that is benchmarked to a null model ignoring all patient specific information and simply predicts the empirical prevalence to each patient [13]. A perfect model has an IPA of 100%, a non-informative model has an IPA of 0% and a negative IPA indicates a harmful model.

```
# Rcode corresponding to file ./R/analysis.R

  # evaluate IPA using riskRegression package
  IPA <- riskRegression::IPA( mod, # mod_exp or mod_cox as above
              formula = Surv( time_event, event) ~ 1,
              newdata = implementation_data,
              times = t_val)$IPA[2]
```

1.6 Software

The simulation study was performed using R statistical software version 3.6.3 [14]. The simulation code is available from `https://github.com/KLuijken/PMH_Survival` and is structured according to the `targets` package [15]. The most important dependencies are the `survival` package for the survival functionalities and fitting the cox regression model [16], `rms` package for fitting the parameteric survival model [17], `pec` package for predicting survival risks [18], `timeROC` package [19] for estimating the $AUC(t)$, and `riskRegression` package [20] for estimating the IPA. The simulation design was described according to Morris and colleagues [21].

```
## R version 3.6.3 (2020-02-29)
## Platform: x86_64-w64-mingw32/x64 (64-bit)
## Running under: Windows 10 x64 (build 19043)
##
## Matrix products: default
##
## locale:
## [1] LC_COLLATE=Dutch_Netherlands.1252  LC_CTYPE=Dutch_Netherlands.1252
## [3] LC_MONETARY=Dutch_Netherlands.1252 LC_NUMERIC=C
## [5] LC_TIME=Dutch_Netherlands.1252
##
## attached base packages:
## character(0)
##
## other attached packages:
## [1] targets_0.3.1            tarchetypes_0.1.1
## [3] survival_3.2-10          rms_6.2-0
## [5] pec_2020.11.17           timeROC_0.4
## [7] riskRegression_2020.12.08
##
## loaded via a namespace (and not attached):
##  [1] Rcpp_1.0.6        svglite_2.0.0     lubridate_1.7.10  tidyr_1.1.2
##  [5] ps_1.6.0          assertthat_0.2.1  digest_0.6.27     utf8_1.1.4
##  [9] R6_2.5.0          cellranger_1.1.0  backports_1.2.1   reprex_2.0.0
## [13] evaluate_0.14     httr_1.4.2        ggplot2_3.3.5     pillar_1.6.0
## [17] utils_3.6.3       rlang_0.4.10      readxl_1.3.1      rstudioapi_0.13
## [21] data.table_1.14.0 callr_3.6.0       rticles_0.20      rmarkdown_2.9
## [25] webshot_0.5.2     readr_1.4.0       stringr_1.4.0     igraph_1.2.6
## [29] munsell_0.5.0     broom_0.7.6       compiler_3.6.3    modelr_0.1.8
## [33] xfun_0.22         pkgconfig_2.0.3   stats_3.6.3       systemfonts_1.0.1
## [37] htmltools_0.5.1.1 tidyselect_1.1.0  tibble_3.0.6      codetools_0.2-16
## [41] grDevices_3.6.3   fansi_0.4.2       viridisLite_0.4.0 crayon_1.4.1
## [45] dplyr_1.0.4       dbplyr_2.1.1      withr_2.4.2       grid_3.6.3
## [49] jsonlite_1.7.2    gtable_0.3.0      lifecycle_1.0.0   DBI_1.1.1
## [53] magrittr_2.0.1    datasets_3.6.3    scales_1.1.1      cli_3.0.1
## [57] stringi_1.5.3     fs_1.5.0          tidyverse_1.3.0   xml2_1.3.2
## [61] ellipsis_0.3.1    graphics_3.6.3    generics_0.1.0    vctrs_0.3.6
## [65] base_3.6.3        kableExtra_1.3.4  tools_3.6.3       forcats_0.5.1
## [69] glue_1.4.2        purrr_0.3.4       hms_1.0.0         processx_3.5.0
## [73] yaml_2.2.1        colorspace_2.0-0  rvest_1.0.0       knitr_1.33
## [77] haven_2.3.1       methods_3.6.3
```

2 Results of simulation study

Additional to the results presented in the main text, we will present descriptive results, to facilitate replication of the simulation study.

2.1 Descriptives

Derivation data (3 censoring mechanisms)

```
## $no_censoring
##    time_event         event         X                   W            
##  Min.   :  0.000   Min.   :1   Min.   :-4.909710   Min.   :-4.909710  
##  1st Qu.:  2.455   1st Qu.:1   1st Qu.:-0.674209   1st Qu.:-0.674209  
##  Median :  6.574   Median :1   Median : 0.000487   Median : 0.000487  
##  Mean   : 12.715   Mean   :1   Mean   : 0.000220   Mean   : 0.000220  
##  3rd Qu.: 15.377   3rd Qu.:1   3rd Qu.: 0.676339   3rd Qu.: 0.676339  
##  Max.   :914.211   Max.   :1   Max.   : 4.963991   Max.   : 4.963991  
## 
## $administrative
##    time_event           event              X                   W            
##  Min.   : 0.000016   Min.   :0.0000   Min.   :-4.909710   Min.   :-4.909710  
##  1st Qu.: 2.454830   1st Qu.:0.0000   1st Qu.:-0.674209   1st Qu.:-0.674209  
##  Median : 6.574485   Median :1.0000   Median : 0.000487   Median : 0.000487  
##  Mean   : 7.681731   Mean   :0.7433   Mean   : 0.000220   Mean   : 0.000220  
##  3rd Qu.:15.000000   3rd Qu.:1.0000   3rd Qu.: 0.676339   3rd Qu.: 0.676339  
##  Max.   :15.000000   Max.   :1.0000   Max.   : 4.963991   Max.   : 4.963991  
## 
## $random
##    time_event           event              X                   W            
##  Min.   : 0.000016   Min.   :0.0000   Min.   :-4.909710   Min.   :-4.909710  
##  1st Qu.: 2.113660   1st Qu.:0.0000   1st Qu.:-0.674209   1st Qu.:-0.674209  
##  Median : 5.571124   Median :1.0000   Median : 0.000487   Median : 0.000487  
##  Mean   : 7.010540   Mean   :0.6854   Mean   : 0.000220   Mean   : 0.000220  
##  3rd Qu.:12.685845   3rd Qu.:1.0000   3rd Qu.: 0.676339   3rd Qu.: 0.676339  
##  Max.   :15.000000   Max.   :1.0000   Max.   : 4.963991   Max.   : 4.963991
```

Validation data (3 censoring mechanisms)

```
##  censoring_mechanism time_event_mean event_mean       X_mean       W_mean
##         no_censoring       12.714650   1.000000 0.0009515512 0.0009515512
##       administrative        7.679506   0.743456 0.0009515512 0.0009515512
##               random        7.010883   0.685331 0.0009515512 0.0009515512
##  time_event_median event_median   X_median   W_median time_event_sd  event_sd
##           6.569671            1 0.00121258 0.00121258     18.968453 0.0000000
##           6.569671            1 0.00121258 0.00121258      5.534070 0.4367257
##           5.568162            1 0.00121258 0.00121258      5.385947 0.4643841
##      X_sd     W_sd
##  1.000799 1.000799
##  1.000799 1.000799
##  1.000799 1.000799
```

Implementation data (3 censoring mechanisms) See end of document.

2.2 Prediction models

2.2.1 Parametric exponential survival model

```
## $no_censoring
## (Intercept)           X 
##   2.3011748  -0.6946524 
## 
## $administrative
## (Intercept)           X 
##   2.3027827  -0.6962639 
## 
```

```
## $random
## (Intercept)           X
##    2.302805   -0.696730
```

2.2.2 Semi-parametric Cox model

```
## $no_censoring
##         X
## 0.6958502
##
## $administrative
##         X
## 0.6964663
##
## $random
##         X
## 0.6970661
```

2.3 External predictive performance under predictor measurement homogeneity

2.3.1 Parametric exponential survival model

censoring_mechanism	cal_large	c_stat	IPA
no_censoring	1	0.74	0.17
administrative	1	0.74	0.17
random	1	0.74	0.17

Validation of the parametric exponential survival model in the validation data, i.e., under predictor measurement heterogeneity, yielded the following results. Across the three censoring scenarios, the calibration-in-the-large coefficient (a measure of weak calibration) equaled 1, indicating good calibration. The AUC(t = 6.5) ranged from 0.74 to 0.74, indicating a discriminatory ability similar to derivation. The IPA(t = 6.5) ranged from 0.17 to 0.17, indicating an accuracy similar to derivation.

2.3.2 Semi-parametric Cox model

censoring_mechanism	cal_large	c_stat	IPA
no_censoring	1	0.74	0.17
administrative	1	0.74	0.17
random	1	0.74	0.17

Validation of the semi-parametric Cox model in the validation data, i.e., under predictor measurement heterogeneity, yielded the following results. Across the three censoring scenarios, the calibration-in-the-large coefficient (a measure of weak calibration) equaled 1, indicating good calibration. The AUC(t = 6.5) ranged from 0.74 to 0.74, indicating a discriminatory ability similar to derivation. The IPA(t = 6.5) ranged from 0.17 to 0.17, indicating an accuracy similar to derivation.

2.4 External predictive performance under predictor measurement heterogeneity

As measurement procedure W contained more random variability compared to X, i.e., a case of random measurement heterogeneity, $\sigma_\epsilon > 0$, the O/E ratio moved slightly under 1 (Figure 1A). The AUC(t = 6.5) and IPA(t = 6.5) decreased as random measurement heterogeneity increased.

Additive systematic measurement heterogeneity, i.e., $\psi \neq 0$, affected the calibration-in-the-large coefficient at implementation, but minimally affected the AUC(t = 6.5) and IPA(t = 6.5) at implementation (Figure 1B). When measurement procedure W at implementation provided a systematically higher value of the predictor compared to measurement procedure X at validation, i.e., $\psi > 0$, this resulted in overestimation of the average outcome incidence at implementation, and the O/E ratio < 1.

Multiplicative systematic measurement heterogeneity, i.e., $\theta \neq 1$, yielded a negative calibration-in-the-large coefficient in case $\theta > 1$ (Figure 1C). Multiplicative systematic measurement heterogeneity minimally affected

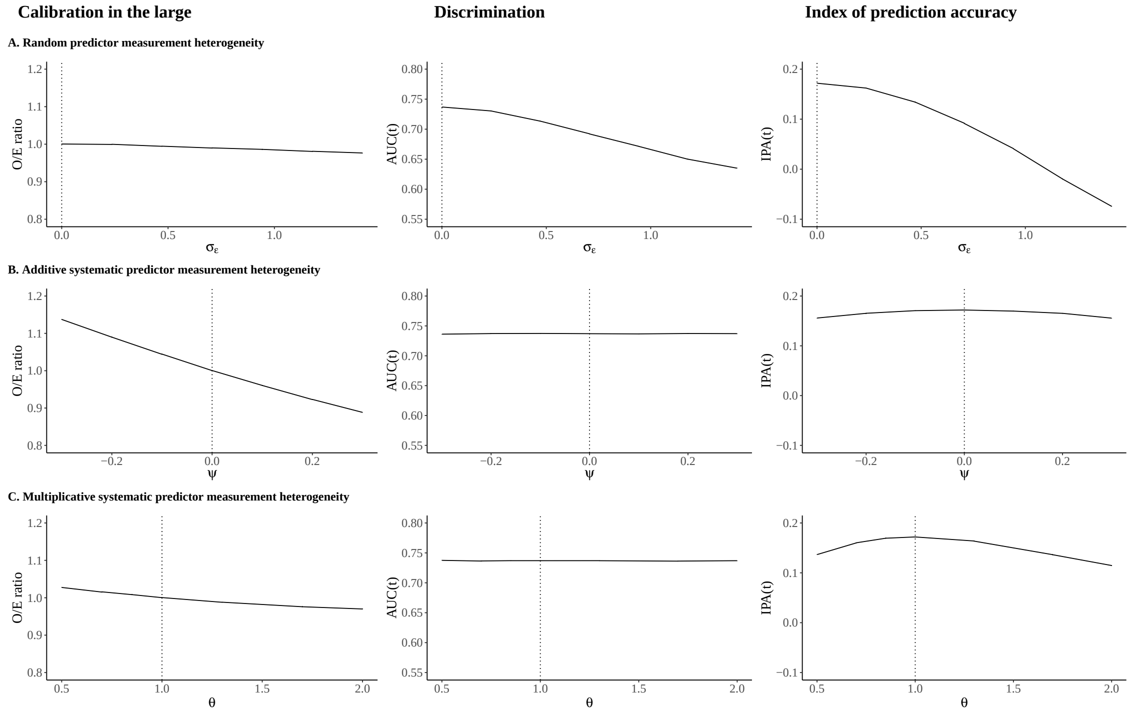

Figure 1: Simulation results external predictive performance under predictor measurement heterogeneity.

the AUC(t = 6.5) in absence of additive systematic and random measurement heterogeneity. As θ was further from 1, the IPA(t = 6.5) at implementation decreased, indicating lower overall accuracy.

3 Detailed results

Measures of predictive performance in all scenarios are presented to illustrate that combined random, additive systematic, and/or multiplicative systematic predictor measurement heterogeneity sometimes reinforced or cancelled out effects on predictive performance. We additionally present descriptives of the simulated implementation datasets to facilitate replication of findings.

3.1 Full results external predictive performance of parametric exponential survival model

3.1.1 No censoring

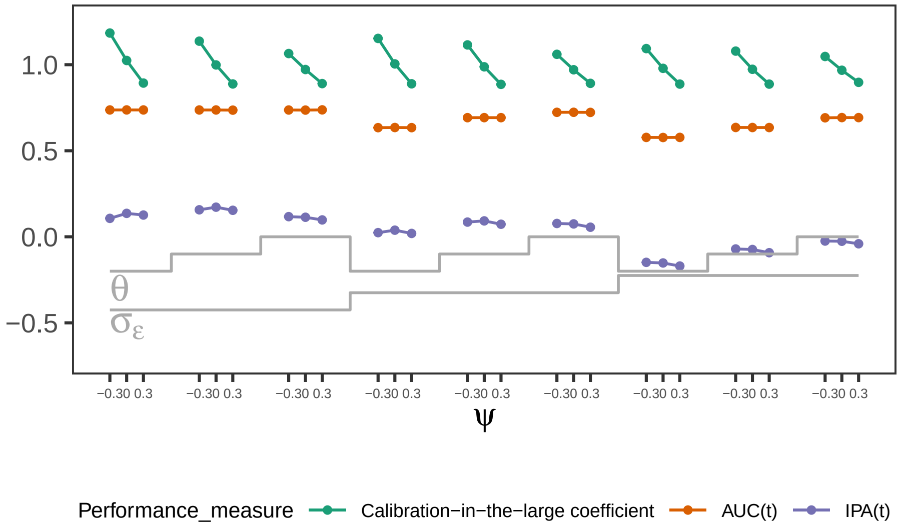

3.1.2 Administrative censoring

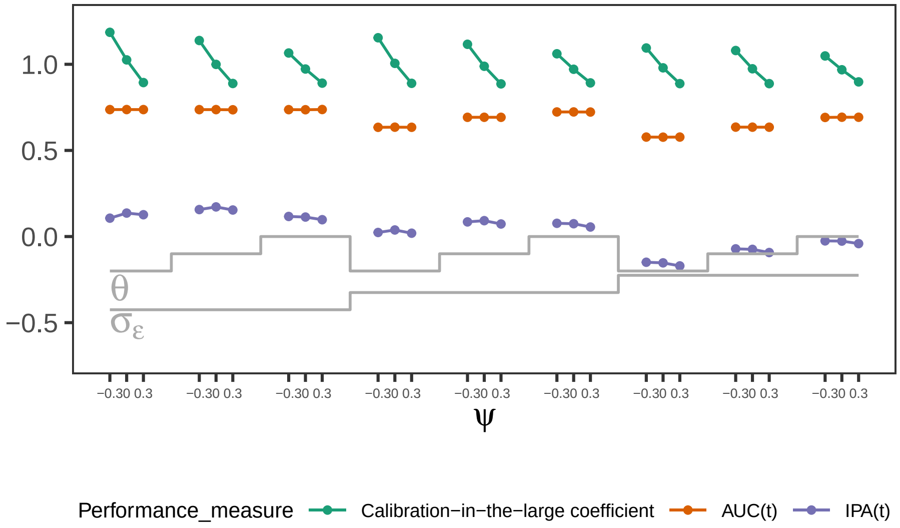

3.1.3 Random censoring

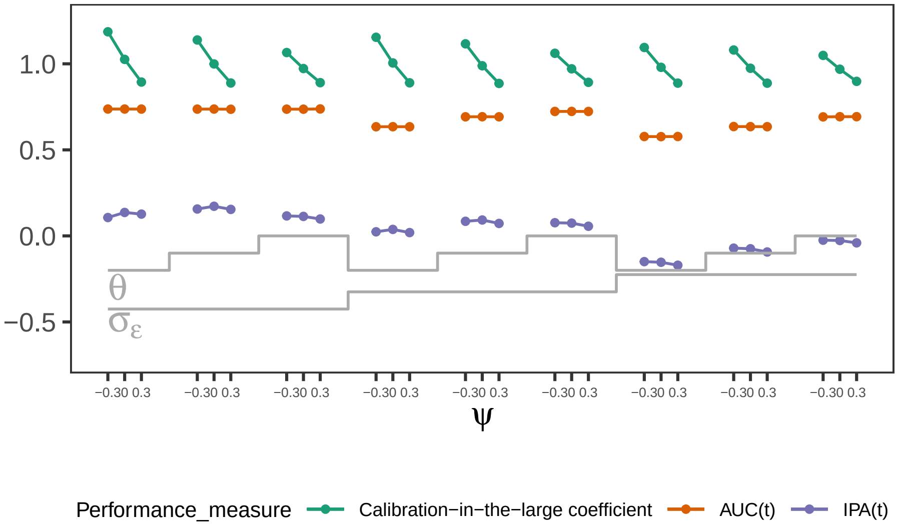

3.2 Full results external predictive performance of semi-parametric Cox survival model

3.2.1 No censoring

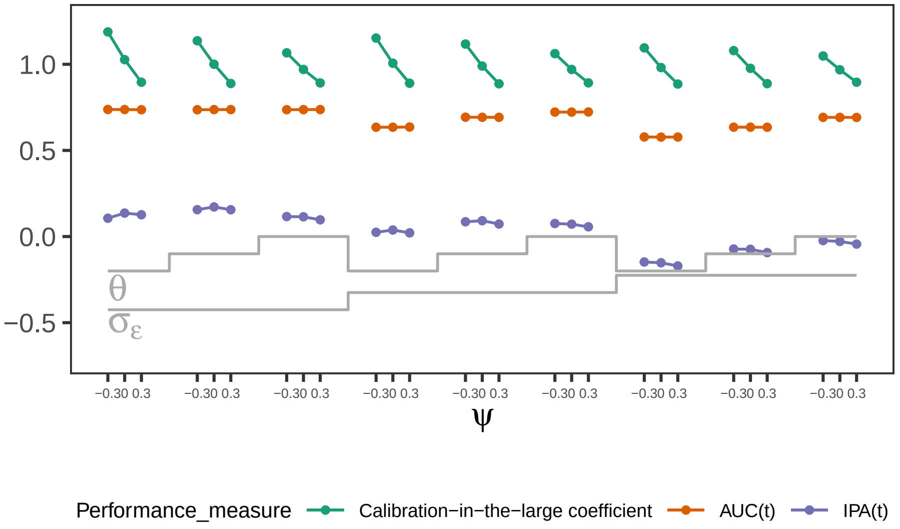

3.2.2 Administrative censoring

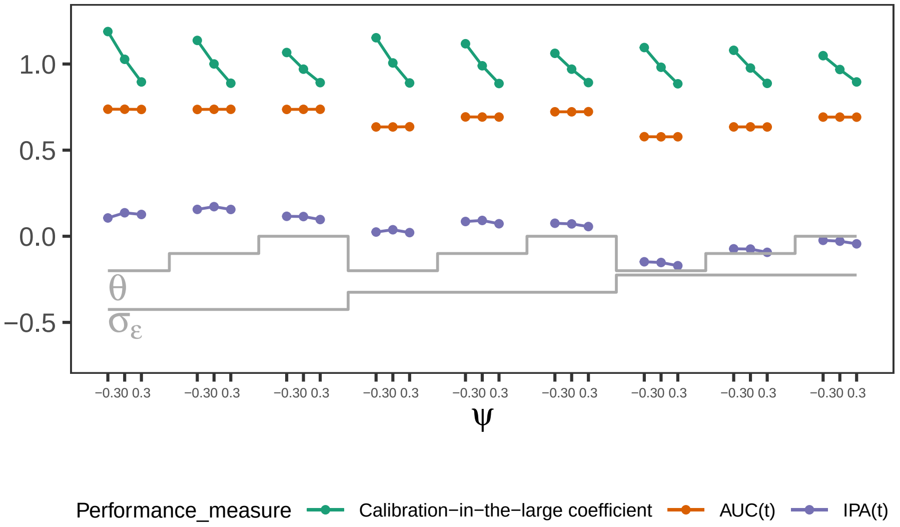

3.2.3 Random censoring

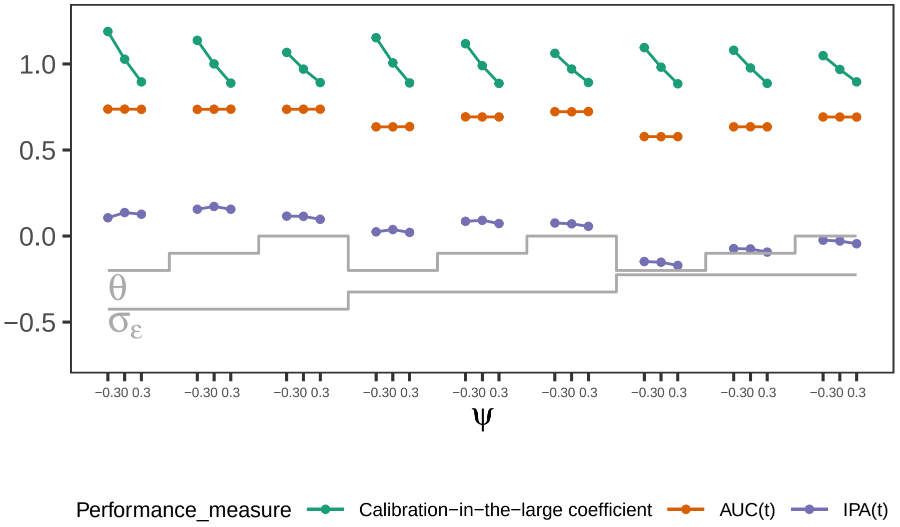

Table 1: Descriptives implementation data.

psi	theta	sigma_epsilon	time_event_mean	event_mean	X_mean	W_mean	time_event_median	event_median	X_median	W_median	time_event_sd	event_sd	X_sd	W_sd
-0.3	0.5	0.00	12.75	1.00	0	-0.3	6.59	1	0	-0.3	19.09	0.00	1	0.50
-0.3	0.5	0.00	7.69	0.74	0	-0.3	6.59	1	0	-0.3	5.53	0.44	1	0.50
-0.3	0.5	0.00	7.02	0.69	0	-0.3	5.58	1	0	-0.3	5.39	0.46	1	0.50
0.0	0.5	0.00	12.71	1.00	0	0.0	6.57	1	0	0.0	18.98	0.00	1	0.50
0.0	0.5	0.00	7.68	0.74	0	0.0	6.57	1	0	0.0	5.53	0.44	1	0.50
0.0	0.5	0.00	7.01	0.69	0	0.0	5.56	1	0	0.0	5.38	0.46	1	0.50
0.3	0.5	0.00	12.75	1.00	0	0.3	6.59	1	0	0.3	19.10	0.00	1	0.50
0.3	0.5	0.00	7.69	0.74	0	0.3	6.59	1	0	0.3	5.54	0.44	1	0.50
0.3	0.5	0.00	7.02	0.68	0	0.3	5.58	1	0	0.3	5.39	0.46	1	0.50
-0.3	1.0	0.00	12.71	1.00	0	-0.3	6.58	1	0	-0.3	19.03	0.00	1	1.00
-0.3	1.0	0.00	7.68	0.74	0	-0.3	6.58	1	0	-0.3	5.53	0.44	1	1.00
-0.3	1.0	0.00	7.01	0.69	0	-0.3	5.57	1	0	-0.3	5.38	0.46	1	1.00
0.3	1.0	0.00	12.71	1.00	0	0.3	6.56	1	0	0.3	19.01	0.00	1	1.00
0.3	1.0	0.00	7.67	0.74	0	0.3	6.56	1	0	0.3	5.53	0.44	1	1.00
0.3	1.0	0.00	7.00	0.69	0	0.3	5.56	1	0	0.3	5.38	0.46	1	1.00
-0.3	2.0	0.00	12.70	1.00	0	-0.3	6.57	1	0	-0.3	18.89	0.00	1	2.00
-0.3	2.0	0.00	7.68	0.74	0	-0.3	6.57	1	0	-0.3	5.54	0.44	1	2.00
-0.3	2.0	0.00	7.01	0.69	0	-0.3	5.57	1	0	-0.3	5.39	0.46	1	2.00
0.0	2.0	0.00	12.73	1.00	0	0.0	6.56	1	0	0.0	19.05	0.00	1	2.00
0.0	2.0	0.00	7.68	0.74	0	0.0	6.56	1	0	0.0	5.53	0.44	1	2.00
0.0	2.0	0.00	7.00	0.69	0	0.0	5.56	1	0	0.0	5.39	0.46	1	2.00
0.3	2.0	0.00	12.73	1.00	0	0.3	6.58	1	0	0.3	19.13	0.00	1	2.00
0.3	2.0	0.00	7.69	0.74	0	0.3	6.58	1	0	0.3	5.53	0.44	1	2.00
0.3	2.0	0.00	7.01	0.68	0	0.3	5.57	1	0	0.3	5.38	0.46	1	2.00
-0.3	0.5	0.71	12.69	1.00	0	-0.3	6.55	1	0	-0.3	19.00	0.00	1	0.87
-0.3	0.5	0.71	7.67	0.74	0	-0.3	6.55	1	0	-0.3	5.53	0.44	1	0.87
-0.3	0.5	0.71	7.00	0.69	0	-0.3	5.55	1	0	-0.3	5.38	0.46	1	0.87
0.0	0.5	0.71	12.71	1.00	0	0.0	6.58	1	0	0.0	18.93	0.00	1	0.86
0.0	0.5	0.71	7.69	0.74	0	0.0	6.58	1	0	0.0	5.53	0.44	1	0.86
0.0	0.5	0.71	7.01	0.69	0	0.0	5.58	1	0	0.0	5.38	0.46	1	0.86

psi	theta	sigma_epsilon	time_event_mean	event_mean	X_mean	W_mean	time_event_median	event_median	X_median	W_median	time_event_sd	event_sd	X_sd	W_sd
0.3	0.5	0.71	12.71	1.00	0	0.3	6.57	1	0	0.30	19.03	0.00	1	0.87
0.3	0.5	0.71	7.68	0.74	0	0.3	6.57	1	0	0.30	5.53	0.44	1	0.87
0.3	0.5	0.71	7.00	0.69	0	0.3	5.56	1	0	0.30	5.38	0.46	1	0.87
-0.3	1.0	0.71	12.74	1.00	0	-0.3	6.58	1	0	-0.30	19.04	0.00	1	1.23
-0.3	1.0	0.71	7.69	0.74	0	-0.3	6.58	1	0	-0.30	5.53	0.44	1	1.23
-0.3	1.0	0.71	7.01	0.69	0	-0.3	5.58	1	0	-0.30	5.38	0.46	1	1.23
0.0	1.0	0.71	12.74	1.00	0	0.0	6.58	1	0	0.00	19.09	0.00	1	1.22
0.0	1.0	0.71	7.69	0.74	0	0.0	6.58	1	0	0.00	5.54	0.44	1	1.22
0.0	1.0	0.71	7.02	0.68	0	0.0	5.57	1	0	0.00	5.39	0.46	1	1.22
0.3	1.0	0.71	12.71	1.00	0	0.3	6.57	1	0	0.30	18.99	0.00	1	1.22
0.3	1.0	0.71	7.69	0.74	0	0.3	6.57	1	0	0.30	5.54	0.44	1	1.22
0.3	1.0	0.71	7.02	0.68	0	0.3	5.57	1	0	0.30	5.39	0.46	1	1.22
-0.3	2.0	0.71	12.71	1.00	0	-0.3	6.56	1	0	-0.30	18.97	0.00	1	2.12
-0.3	2.0	0.71	7.67	0.74	0	-0.3	6.56	1	0	-0.30	5.53	0.44	1	2.12
-0.3	2.0	0.71	7.00	0.69	0	-0.3	5.55	1	0	-0.30	5.38	0.46	1	2.12
0.0	2.0	0.71	12.73	1.00	0	0.0	6.56	1	0	-0.01	19.08	0.00	1	2.12
0.0	2.0	0.71	7.68	0.74	0	0.0	6.56	1	0	-0.01	5.54	0.44	1	2.12
0.0	2.0	0.71	7.01	0.69	0	0.0	5.56	1	0	-0.01	5.39	0.46	1	2.12
0.3	2.0	0.71	12.73	1.00	0	0.3	6.58	1	0	0.30	18.97	0.00	1	2.12
0.3	2.0	0.71	7.68	0.74	0	0.3	6.58	1	0	0.30	5.53	0.44	1	2.12
0.3	2.0	0.71	7.01	0.68	0	0.3	5.57	1	0	0.30	5.38	0.46	1	2.12
-0.3	0.5	1.41	12.74	1.00	0	-0.3	6.58	1	0	-0.30	19.04	0.00	1	1.50
-0.3	0.5	1.41	7.69	0.74	0	-0.3	6.58	1	0	-0.30	5.54	0.44	1	1.50
-0.3	0.5	1.41	7.01	0.69	0	-0.3	5.57	1	0	-0.30	5.39	0.46	1	1.50
0.0	0.5	1.41	12.72	1.00	0	0.0	6.59	1	0	0.00	18.91	0.00	1	1.50
0.0	0.5	1.41	7.69	0.74	0	0.0	6.59	1	0	0.00	5.53	0.44	1	1.50
0.0	0.5	1.41	7.02	0.69	0	0.0	5.57	1	0	0.00	5.38	0.46	1	1.50
0.3	0.5	1.41	12.71	1.00	0	0.3	6.57	1	0	0.30	19.00	0.00	1	1.50
0.3	0.5	1.41	7.68	0.74	0	0.3	6.57	1	0	0.30	5.53	0.44	1	1.50
0.3	0.5	1.41	7.00	0.69	0	0.3	5.56	1	0	0.30	5.39	0.46	1	1.50

psi	theta	sigma_epsilon	time_event_mean	event_mean	X_mean	W_mean	time_event_median	event_median	X_median	W_median	time_event_sd	event_sd	X_sd	W_sd
-0.3	1	1.41	12.70	1.00	0	-0.3	6.57	1	0	-0.3	18.88	0.00	1	1.73
-0.3	1	1.41	7.68	0.74	0	-0.3	6.57	1	0	-0.3	5.54	0.44	1	1.73
-0.3	1	1.41	7.01	0.69	0	-0.3	5.56	1	0	-0.3	5.39	0.46	1	1.73
0.0	1	1.41	12.71	1.00	0	0.0	6.58	1	0	0.0	19.05	0.00	1	1.73
0.0	1	1.41	7.69	0.74	0	0.0	6.58	1	0	0.0	5.53	0.44	1	1.73
0.0	1	1.41	7.02	0.69	0	0.0	5.58	1	0	0.0	5.38	0.46	1	1.73
0.3	1	1.41	12.71	1.00	0	0.3	6.59	1	0	0.3	18.96	0.00	1	1.73
0.3	1	1.41	7.69	0.74	0	0.3	6.59	1	0	0.3	5.53	0.44	1	1.73
0.3	1	1.41	7.01	0.69	0	0.3	5.58	1	0	0.3	5.38	0.46	1	1.73
-0.3	2	1.41	12.69	1.00	0	-0.3	6.56	1	0	-0.3	18.91	0.00	1	2.45
-0.3	2	1.41	7.68	0.74	0	-0.3	6.56	1	0	-0.3	5.54	0.44	1	2.45
-0.3	2	1.41	7.01	0.69	0	-0.3	5.55	1	0	-0.3	5.39	0.46	1	2.45
0.0	2	1.41	12.71	1.00	0	0.0	6.55	1	0	0.0	19.00	0.00	1	2.45
0.0	2	1.41	7.67	0.74	0	0.0	6.55	1	0	0.0	5.53	0.44	1	2.45
0.0	2	1.41	7.00	0.69	0	0.0	5.55	1	0	0.0	5.38	0.46	1	2.45
0.3	2	1.41	12.71	1.00	0	0.3	6.55	1	0	0.3	19.04	0.00	1	2.45
0.3	2	1.41	7.67	0.74	0	0.3	6.55	1	0	0.3	5.53	0.44	1	2.45
0.3	2	1.41	7.00	0.69	0	0.3	5.56	1	0	0.3	5.38	0.46	1	2.45
-0.2	1	0.00	12.71	1.00	0	-0.2	6.58	1	0	-0.2	19.00	0.00	1	1.00
-0.2	1	0.00	7.68	0.74	0	-0.2	6.58	1	0	-0.2	5.54	0.44	1	1.00
-0.2	1	0.00	7.01	0.69	0	-0.2	5.56	1	0	-0.2	5.39	0.46	1	1.00
-0.1	1	0.00	12.70	1.00	0	-0.1	6.57	1	0	-0.1	18.95	0.00	1	1.00
-0.1	1	0.00	7.68	0.74	0	-0.1	6.57	1	0	-0.1	5.53	0.44	1	1.00
-0.1	1	0.00	7.01	0.69	0	-0.1	5.57	1	0	-0.1	5.38	0.46	1	1.00
0.1	1	0.00	12.74	1.00	0	0.1	6.58	1	0	0.1	19.02	0.00	1	1.00
0.1	1	0.00	7.69	0.74	0	0.1	6.58	1	0	0.1	5.53	0.44	1	1.00
0.1	1	0.00	7.01	0.69	0	0.1	5.57	1	0	0.1	5.39	0.46	1	1.00
0.2	1	0.00	12.71	1.00	0	0.2	6.57	1	0	0.2	19.04	0.00	1	1.00
0.2	1	0.00	7.67	0.74	0	0.2	6.57	1	0	0.2	5.53	0.44	1	1.00
0.2	1	0.00	7.00	0.69	0	0.2	5.55	1	0	0.2	5.38	0.46	1	1.00

**psi**	**theta**	**sigma_epsilon**	**time_event_mean**	**event_mean**	**X_mean**	**W_mean**	**time_event_median**	**event_median**	**X_median**	**W_median**	**time_event_sd**	**event_sd**	**X_sd**	**W_sd**
0	0.70	0.00	12.68	1.00	0	0	6.54	1	0	0	18.96	0.00	1	0.70
0	0.70	0.00	7.67	0.74	0	0	6.54	1	0	0	5.53	0.44	1	0.70
0	0.70	0.00	7.00	0.69	0	0	5.55	1	0	0	5.38	0.46	1	0.70
0	0.85	0.00	12.66	1.00	0	0	6.54	1	0	0	18.96	0.00	1	0.85
0	0.85	0.00	7.67	0.74	0	0	6.54	1	0	0	5.53	0.44	1	0.85
0	0.85	0.00	7.00	0.69	0	0	5.55	1	0	0	5.38	0.46	1	0.85
0	1.30	0.00	12.68	1.00	0	0	6.54	1	0	0	18.94	0.00	1	1.30
0	1.30	0.00	7.66	0.74	0	0	6.54	1	0	0	5.53	0.44	1	1.30
0	1.30	0.00	7.00	0.69	0	0	5.55	1	0	0	5.38	0.46	1	1.30
0	1.70	0.00	12.69	1.00	0	0	6.56	1	0	0	19.00	0.00	1	1.70
0	1.70	0.00	7.67	0.74	0	0	6.56	1	0	0	5.53	0.44	1	1.70
0	1.70	0.00	7.00	0.69	0	0	5.55	1	0	0	5.38	0.46	1	1.70
0	1.00	0.24	12.72	1.00	0	0	6.56	1	0	0	19.10	0.00	1	1.03
0	1.00	0.24	7.68	0.74	0	0	6.56	1	0	0	5.53	0.44	1	1.03
0	1.00	0.24	7.01	0.69	0	0	5.56	1	0	0	5.38	0.46	1	1.03
0	1.00	0.47	12.73	1.00	0	0	6.57	1	0	0	19.04	0.00	1	1.11
0	1.00	0.47	7.68	0.74	0	0	6.57	1	0	0	5.54	0.44	1	1.11
0	1.00	0.47	7.01	0.69	0	0	5.56	1	0	0	5.39	0.46	1	1.11
0	1.00	0.94	12.70	1.00	0	0	6.57	1	0	0	18.97	0.00	1	1.37
0	1.00	0.94	7.68	0.74	0	0	6.57	1	0	0	5.53	0.44	1	1.37
0	1.00	0.94	7.01	0.68	0	0	5.56	1	0	0	5.39	0.46	1	1.37
0	1.00	1.18	12.74	1.00	0	0	6.58	1	0	0	19.08	0.00	1	1.55
0	1.00	1.18	7.69	0.74	0	0	6.58	1	0	0	5.54	0.44	1	1.55
0	1.00	1.18	7.02	0.68	0	0	5.58	1	0	0	5.39	0.46	1	1.55

References

1. Bender R, Augustin T, Blettner M. Generating survival times to simulate cox proportional hazards models. Statistics in medicine. 2005;24:1713–23.

2. Luijken K, Groenwold RH, Van Calster B, Steyerberg EW, Smeden M van. Impact of predictor measurement heterogeneity across settings on the performance of prediction models: A measurement error perspective. Statistics in medicine. 2019;38:3444–59.

3. Keogh RH, White IR. A toolkit for measurement error correction, with a focus on nutritional epidemiology. Statistics in medicine. 2014;33:2137–55.

4. Carroll RJ, Ruppert D, Stefanski LA, Crainiceanu CM. Measurement error in nonlinear models: A modern perspective. CRC press; 2006.

5. Harrell Jr FE, Lee KL, Mark DB. Multivariable prognostic models: Issues in developing models, evaluating assumptions and adequacy, and measuring and reducing errors. Statistics in medicine. 1996;15:361–87.

6. Steyerberg EW, others. Clinical prediction models. Springer; 2019.

7. Van Calster B, Nieboer D, Vergouwe Y, De Cock B, Pencina MJ, Steyerberg EW. A calibration hierarchy for risk models was defined: From utopia to empirical data. Journal of clinical epidemiology. 2016;74:167–76.

8. Van Calster B, McLernon DJ, Van Smeden M, Wynants L, Steyerberg EW. Calibration: The achilles heel of predictive analytics. BMC medicine. 2019;17:1–7.

9. Heagerty PJ, Zheng Y. Survival model predictive accuracy and roc curves. Biometrics. 2005;61:92–105.

10. Uno H, Cai T, Tian L, Wei L-J. Evaluating prediction rules for t-year survivors with censored regression models. Journal of the American Statistical Association. 2007;102:527–37.

11. Blanche P, Kattan MW, Gerds TA. The c-index is not proper for the evaluation of-year predicted risks. Biostatistics. 2019;20:347–57.

12. Brier GW. Verification of forecasts expressed in terms of probability. Monthly weather review. 1950;78:1–3.

13. Kattan MW, Gerds TA. The index of prediction accuracy: An intuitive measure useful for evaluating risk prediction models. Diagnostic and prognostic research. 2018;2:1–7.

14. R Core Team. R: A language and environment for statistical computing. Vienna, Austria: R Foundation for Statistical Computing; 2020. `https://www.R-project.org/`.

15. Landau WM. The targets r package: A dynamic make-like function-oriented pipeline toolkit for reproducibility and high-performance computing. Journal of Open Source Software. 2021;6:2959. `https://doi.org/10.21105/joss.02959`.

16. Therneau TM. A package for survival analysis in r. 2021. `https://CRAN.R-project.org/package=survival`.

17. Harrell Jr FE. Rms: Regression modeling strategies. 2021. `https://CRAN.R-project.org/package=rms`.

18. Mogensen UB, Ishwaran H, Gerds TA. Evaluating random forests for survival analysis using prediction error curves. Journal of Statistical Software. 2012;50:1–23. `https://www.jstatsoft.org/v50/i11`.

19. Blanche P, Dartigues J-F, Jacqmin-Gadda H. Estimating and comparing time-dependent areas under receiver operating characteristic curves for censored event times with competing risks. Statistics in Medicine. 2013;32:5381–97. `http://onlinelibrary.wiley.com/doi/10.1002/sim.5958/full`.

20. Gerds TA, Ozenne B. RiskRegression: Risk regression models and prediction scores for survival analysis with competing risks. 2020. `https://CRAN.R-project.org/package=riskRegression`.

21. Morris TP, White IR, Crowther MJ. Using simulation studies to evaluate statistical methods. Statistics in medicine. 2019;38:2074–102.
