# Supplementary material for: Quantitative prediction error analysis to investigate predictive performance under predictor measurement heterogeneity at model implementation
Source: Diagn Progn Res. 2022 Apr 7;6:7. doi: 10.1186/s41512-022-00121-1 (PMC8988417; doi:10.1186/s41512-022-00121-1)
Supplement: Supplementary file 2 — Additional file 2. Sensitivity analysis assessing the impact of anticipated predictor measurement heterogeneity across validation and implementation setting in time-to-event outcome data. An example on prediction of incident diabetes type 2. [file 41512_2022_121_MOESM2_ESM.pdf]

Sensitivity analysis assessing the impact of anticipated predictor measurement heterogeneity across validation and implementation setting in time-to-event outcome data. An example on prediction of incident diabetes type 2.

Supplementary File 2

Kim Luijken
Department of Clinical Epidemiology
Leiden University Medical Center

k.luijken@lumc.nl

Jia Song
Department of Clinical Epidemiology
Leiden University Medical Center

Rolf Groenwold
Department of Clinical Epidemiology
Leiden University Medical Center

December 22, 2021

Content

This Supplementary File accompanies the manuscript "Quantitative prediction error analysis to investigate predictive performance under predictor measurement heterogeneity at model implementation" by Kim Luijken, Jia Song, and Rolf Groenwold. It contains a description of the sensitivity analysis described in Section 4 of the main text.

1 Validation of prediction model

Zhang and colleagues derived a prognostic model predicting the 6-year risk of developing type-2 diabetes from the predictors age, BMI, triglyceride, and fasting plasma glucose at moment of prediction[1].

Comparison of derivation and validation cohort

The derivation cohort consisted of adults recruited from a rural Chinese population from July to August of 2007 and July to August of 2008 (baseline). The validation cohort consisted of adults who participated in a medical examination program at the Murakami Memorial Hospital (Japan) from 2004 to 2015.
The cohorts were compared on the predictor variables and outcome (in an actual validation study it is recommended to extend this comparison to other characteristics):

Characteristic	Derivation	Validation
Age (years), median (IQR)	51 (42,59)	42 (37,50)
BMI (kg/m2), median (IQR)	24.09 (21.76, 26.59)	21.79 (19.89, 23.92)
FPG (mmol/L), median (IQR)	5.32 (4.99, 5.68)	5.16 (4.89, 5.44
TG (mmol/L), median (IQR)	1.35 (0.96, 1.95)	0.73 (0.50, 1.12)
Cases of diabetes in 6 years, incidence/1000 personyears	9.57	2.84

Both cohorts represent an adult Asian general population. Of note, the intended moment of use of the prediction model was somewhat unclear in the derivation study, while for the validation study the presentation at the medical examination program was a natural moment to predict the 6-year risk of diabetes. The incidence of diabetes was lower in the validation cohort, as well as the average age, BMI, fasting plasma glucose, and triglyceride. Despite that, we assume the validation cohort to be similar enough to perform a validation of the prediction model without changing the prediction target (that is, the specified outcome, (candidate) predictors, population, setting, time of prediction, and prediction horizon).

Model validation

When we validated the prediction model as-is, the calibration-in-the-large O/E ratio was 0.47 (95% CI, 0.41 to 0.54), indicating that predicted risks were overestimated on average. The AUC(6 years) was 0.89 (95% CI, 0.85 to 0.89), indicating good discriminatory performance of the model. the IPA(6 years) was 0.02 (95% CI, 0.01 to 0.03), indicating low overall accuracy of the model.

Recalibrate baseline survival

Given the suboptimal calibration of the model and the difference in outcome incidence between derivation and validation setting, we updated the baseline survival at six years by re-estimating it using an offset for the linear predictor. The updated baseline survival at six years was 0.9999957 compared to 0.999998 in the derivation study. Predictive performance of the model after recalibrating the baseline survival was as follows: the calibration-in-the-large O/E ratio was 1.02 (95% CI, 0.90 to 1.18), the AUC(6 years) was 0.87 (95% CI, 0.85 to 0.89), and the IPA(6 years) was 0.04 (95% CI, 0.04 to 0.05).

2 Introduction of the quantitative prediction error analysis

We describe seven steps to perform a quantitative prediction error analysis in a prognostic model validation study to assess the impact of anticipated measurement heterogeneity in measurement of BMI, where BMI is assumed to be measured from self-reported height and weight at implementation, instead of tape and scale measures at validation (summarized in Box 1 of the main text).

Step 1: state the prediction target

In this example, the prediction target would be the 6-year risk of developing adult-onset type-2 diabetes in Asian individuals presenting for preventive medical examination by measurements of age, BMI, triglyceride, and fasting plasma glucose at moment of prediction. Incident diabetes is defined as HbA1c $\geq$ 6.5% (48 mmol/mol) (48 mmol/mol) in two test results, measured using a standardized method [2]. Age is measured in years, BMI is calculated from self-reported weight and height, triglyceride is measured according to standards

of the National Institute of Standards and Technology [3], and fasting plasma glucose is measured using a standardized method [4, 5]. Details on procedures to measure HbA1c, triglyceride, and fasting plasma glucose are omitted here for brevity, but are ideally described in more detail in an empirical study. Treatment assignment policy was assumed to be similar in the research settings compared to the target clinical setting and interventions such as diet were not modeled explicitly (i.e., ignore-treatment strategy [6]).

Step 2: report whether predictor measurement procedures in the derivation and validation setting correspond to the prediction target

Measurements of age, triglyceride, and fasting plasma glucose roughly correspond to the target predictor measurement procedures. However, the validation study measured BMI during medical examination of a patient, which differs from self-reported measurements defined in the prediction target.

Step 3: identify one predictor that is expected to be measured using a different procedure in the implementation setting than in the validation setting

Measurement heterogeneity was expected to be strongest for the predictor BMI.

Step 4: define a model for the relation between the measurement in the validation study and its equivalent in the implementation setting

We start out with a general model allowing for non-differential systematic and random predictor measurement heterogeneity, that is

$$BMI_{imp} = \psi + \theta BMI_{val} + \epsilon,$$

where $\epsilon \sim N(0, \sigma_\epsilon^2)$, and $\psi \neq 0$indicates that measurements of BMI in the implementation setting are systematically additively shifted with respect to BMI in the validation study, $\theta \neq 1$ indicates measurements of BMI in the implementation setting are systematically multiplicatively altered with respect to BMI in the validation study, and $\sigma_\epsilon > 0$ indicates measurements of BMI in the implementation setting contain more random variation relative to BMI in the validation study.

Step 5: perform a literature search to establish a range for the size of the possible parameters of predictor measurement heterogeneity

The range is specified for the parameter values of the model for the anticipated predictor measurement heterogeneity, as defined in Step four. A literature search was performed to identify studies describing measurement error in BMI. Informed by studies comparing measured and self-reported BMI values [7–10], the range of measurement error parameters was specified as -1 to 0 for ψ, 0.9 to 1 for θ, and 0 to 1.5 for σ_ϵ. In general, we advise to use terms like ‘measurement error’, ‘validation study’, and the measurement procedures to search for relevant literature. Of note, the term ‘validation study’ has a different meaning in prediction literature compared to measurement error literature. In prediction literature, a validation study refers to a study that evaluates the predictive performance of an existing prediction model. In measurement error literature, a validation study refers to a study in which a perfect measurement is taken of a mismeasured covariate, usually in a subset of individuals included in the study. The purpose of a measurement-error validation study is to estimate the connection between the error-prone and error-free measurement, for instance using measurement error models, to address issues introduced by measurement error in the substantive analysis. In the current study, we thus far used the term ‘validation study’ according to the prediction literature.

Step 6: simulate the scenarios of anticipated measurement heterogeneity to assess the possible impact on predictive performance

We simulated the scenarios of anticipated measurement heterogeneity to assess the possible impact on predictive performance. R scripts to perform this analysis are available from `https://github.com/KLuijken/PMH_Survival/sensitivity_analysis`. Here, we explain how to apply the R scripts.

Start by cloning the repository or opening an R project with a ./data folder with the validation study dataset and an ./R folder with the sensitivity analysis R scripts. The analysis can be run using file ./R/sensitivity_analysis.R, which calls the data and helper scripts in ./R/helper_functions.R.

Load validation data
We used the file ./R/clean_data.R to prepare the analysis dataset. When preparing your own data file, make sure that the variable with event times and censoring times is named 'time' and the variable with event status and censoring status is named 'event' and takes values 0 and 1.

```
# execute script to clean the data
source( here( "R/clean_data.R"))

# describe validation data set
summary( diabetes_data)
```

```
##       age             bmi               tg                fpg
##  Min.   :18.00   Min.   :13.77   Min.   : 0.06774   Min.   :2.831
##  1st Qu.:37.00   1st Qu.:19.89   1st Qu.: 0.49676   1st Qu.:4.885
##  Median :42.00   Median :21.79   Median : 0.73385   Median :5.162
##  Mean   :43.71   Mean   :22.12   Mean   : 0.91213   Mean   :5.161
##  3rd Qu.:50.00   3rd Qu.:23.92   3rd Qu.: 1.11771   3rd Qu.:5.440
##  Max.   :79.00   Max.   :49.92   Max.   :10.27390   Max.   :6.051
##       time             event
##  Min.   : 164.0   Min.   :0.00000
##  1st Qu.: 986.8   1st Qu.:0.00000
##  Median :1967.0   Median :0.00000
##  Mean   :1595.4   Mean   :0.01242
##  3rd Qu.:2192.0   3rd Qu.:0.00000
##  Max.   :2192.0   Max.   :1.00000
```

Input information on prediction model
Specify the time point at which the model is validated, the predictor names, and the coefficients of predictors and the baseline survival at the time point of validation obtained from the derivation study. Of note, the baseline survival noted here is the recalibrated baseline survival.

```
pred_names <- c( "age", "bmi", "fpg", "tg")
pred_coefs <- c( 0.027, 0.124, 0.76, 0.239)
baseline_surv_tval <- 0.9999956
```

Define scenarios of predictor measurement heterogeneity (pmh)
Specify the range of the parameters of the measurement heterogeneity model described in **step 4** using the values identified from literature in **step 5**.

```
psi        <- c( -1, -0.5, 0)
theta      <- c( 0.9, 1)
sd_epsilon <- c( 0, 1, 1.5)

scenarios  <- expand.grid( psi = psi,                # 18 scenarios
                           theta = theta,
                           sd_epsilon = sd_epsilon)
```

Perform validation across pmh scenarios
Here, we explain the functionalities in ./R/helper_functions.R that perform the validation across scenarios of predictor measurement heterogeneity.
Measurements of BMI that can be anticipated in the implementation setting in participants otherwise similar to the validation sample can be generated by plugging in these values into the measurement heterogeneity model. In our example, the function pmh_sensitivity_analysis() does the following:

```
# generate predictor measurement heterogeneity
pmh_data <- diabetes_data
# random measurement heterogeneity in BMI
epsilon <- rnorm( n = nrow( diabetes_data), mean = 0, sd = sd_epsilon)
# systematic and random measurement heterogeneity in BMI (non-differential)
pmh_data$bmi <- psi + theta * diabetes_data$bmi + epsilon
```

Then, predictive performance at implementation is evaluated using the function validate_model(). Measures of predictive performance are the ratio of the observed marginal survival at $t = 6$ years, obtained through a Kaplan-Meier curve, versus the predicted marginal survival at $t = 6$ years, obtained by averaging predicted survival at $t = 6$ years for each individual, (O / E ratio) for calibration in the large, the AUC(t) for discrimination and the IPA($t = 6$ years) for overall accuracy. These measures are estimated as follows:

```
# compute linear predictor
lp <- as.matrix( diabetes_data[ , pred_names]) %*% pred_coefs

# calibration in the large
#-----------------------------------------------------------------------------#
# compute marginal predicted risk
overall_surv <- baseline_surv ^ exp( lp)
pred_risk <- 1 - overall_surv
marginal_pred_risk <- mean( pred_risk)

# observed marginal risk
marginal_obs_risk <- 1 - summary( survfit( Surv( time, event) ~ 1,
                                           data = diabetes_data),
                         times= t_val)$surv

cal_large <- marginal_pred_risk / marginal_obs_risk

# AUC(t_val)
#-----------------------------------------------------------------------------#
c_stat <- unname(
  timeROC::timeROC( T = dataset$time,
                    delta = dataset$event,
                    marker = lp,
                    cause = 1,
                    times = t_val)$AUC[2])
# IPA (t_val)
#-----------------------------------------------------------------------------#
# individual predicted risks
pred_risk   <- 1 - exp(- expected_events)

IPA <- riskRegression::IPA( pred_risk,
                            formula = Surv( time, event) ~ 1,
                            newdata = diabetes_data,
                            times = t_val,
                            cause = 1)$IPA[2]
```

Step 7: report the impact of anticipated predictor measurement heterogeneity on predictive performance in clinical implementation

The impact of anticipated predictor measurement heterogeneity on predictive performance in the implementation setting can be reported in a validation study, accompanied by a description of Steps 1-6. The figure below illustrates the range of the O/E ratio at 6 years, AUC(6 years), and IPA(6 years) under the anticipated measurement heterogeneity of BMI across validation and implementation setting. The findings suggest that model discrimination and overall accuracy are likely minimally affected by the change in measurement of BMI. However, with increasing differences in BMI measurement, model miscalibration increases and predicted risks are more likely to be overestimated on average.

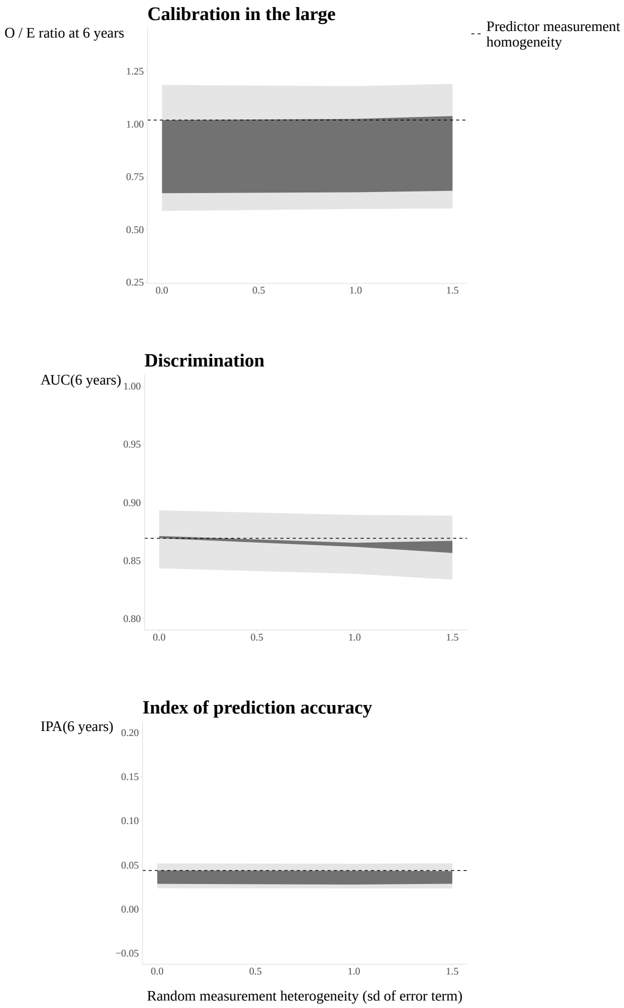

Figure 1: Impact of anticipated heterogeneity in measurement of the predictor BMI on measures of predictive performance at implementation of a model to predict 6-year risk of developing diabetes type 2. The dotted line indicates predictive performance under predictor measurement homogeneity. Dark grey indicates the impact within the range of specified predictor measurement heterogeneity and light grey indicates the range of 95 percentile CIs from 500 bootstrap resamples. Random predictor measurement heterogeneity is presented on the x-axis, and performance measures are marginalized over scenarios of additive and multiplicative systematic predictor measurement heterogeneity.

References

1. Zhang M, Zhang H, Wang C, Ren Y, Wang B, Zhang L, et al. Development and validation of a risk-score model for type 2 diabetes: A cohort study of a rural adult chinese population. Plos one. 2016;11:e0152054.

2. Association AD, others. 2. Classification and diagnosis of diabetes: Standards of medical care in diabetes—2021. Diabetes Care. 2021;44 Supplement 1:S15–33.

3. Warnick GR, Kimberly MM, Waymack PP, Leary ET, Myers GL. Standardization of measurements for cholesterol, triglycerides, and major lipoproteins. Laboratory Medicine. 2008;39:481–90.

4. Organization WH, others. Definition and diagnosis of diabetes mellitus and intermediate hyperglycaemia: Report of a who/idf consultation. 2006.

5. D'Orazio P, Burnett RW, Fogh-Andersen N, Jacobs E, Kuwa K, Külpmann WR, et al. Approved ifcc recommendation on reporting results for blood glucose: International federation of clinical chemistry and laboratory medicine scientific division, working group on selective electrodes and point-of-care testing (ifcc-sd-wg-sepoct). Clinical Chemistry and Laboratory Medicine (CCLM). 2006;44:1486–90.

6. Geloven N van, Swanson SA, Ramspek CL, Luijken K, Diepen M van, Morris TP, et al. Prediction meets causal inference: The role of treatment in clinical prediction models. European journal of epidemiology. 2020;35:619–30.

7. Nawaz H, Chan W, Abdulrahman M, Larson D, Katz DL. Self-reported weight and height: Implications for obesity research. American journal of preventive medicine. 2001;20:294–8.

8. Allison C, Colby S, Opoku-Acheampong A, Kidd T, Kattelmann K, Olfert MD, et al. Accuracy of self-reported bmi using objective measurement in high school students. Journal of Nutritional Science. 2020;9.

9. Dekkers JC, Wier MF van, Hendriksen IJ, Twisk JW, Mechelen W van. Accuracy of self-reported body weight, height and waist circumference in a dutch overweight working population. BMC medical research methodology. 2008;8:1–13.

10. Villarini M, Acito M, Gianfredi V, Berrino F, Gargano G, Somaini M, et al. Validation of self-reported anthropometric measures and body mass index in a subcohort of the dianaweb population study. Clinical breast cancer. 2019;19:e511–8.

11. Ortiz-Panozo E, Yunes-Díaz E, Lajous M, Romieu I, Monge A, López-Ridaura R. Validity of self-reported anthropometry in adult mexican women. Salud publica de Mexico. 2017;59:266–75.
